# Intraocular complement activation is related to retinal vascular and neuronal degeneration in myopic retinopathy

**DOI:** 10.3389/fncel.2023.1187400

**Published:** 2023-06-28

**Authors:** Ling Zeng, Xiaoning Li, Wei Pan, Yao Tang, Ding Lin, Min Wang, Wang Cai, Ruiling Zhu, Jianbo Wan, Linghua Huang, Heping Xu, Zhikuan Yang

**Affiliations:** ^1^Aier School of Ophthalmology, Central South University, Changsha, China; ^2^Changsha Aier Eye Hospital, Changsha, Hunan, China; ^3^Aier Institute of Optometry and Vision Science, Aier Eye Hospital Group, Changsha, China; ^4^Aier School of Optometry and Vision Science, Hubei University of Science and Technology, Xianning, Hubei, China; ^5^Shanghai Aier Eye Hospital, Shanghai, China; ^6^The Wellcome-Wolfson Institute for Experimental Medicine, School of Medicine, Dentistry and Biomedical Sciences, Queen’s University Belfast, Belfast, United Kingdom

**Keywords:** aqueous humour, inflammation, myopic retinal degeneration, posterior staphyloma, myopic chorioretinal atrophy

## Abstract

**Purpose:**

To investigate the relationship between the intraocular levels of complement proteins and myopia-related retinal neuronal and vascular degeneration.

**Methods:**

Aqueous humour from 147 myopic patients, including 60 low-myopia and 87 high-myopia were collected during Implantable Collamer Lens implantation surgery. All participants received comprehensive ophthalmic examinations, including logMAR best corrected visual acuity, axial length measurement, fundus photography and ocular B-scan ultrasonography. The myopic eyes were further classified into simple myopia (SM, *n* = 78), myopic posterior staphyloma (PS, *n* = 39) and PS with myopic chorioretinal atrophy (PS + CA, *n* = 30). Retinal thickness and vascular density in the macula (6 mm × 6 mm) and optic nerve head (4.5 mm × 4.5 mm) were measured using Optical Coherence Tomography (OCT) and OCT angiography (OCTA). The levels of complement proteins including C1q, C3, C3b/iC3b, C4, CFB, CFH, C2, C4b, C5, C5a, CFD, MBL and CFI in the aqueous humour were measured using the Luminex Multiplexing system. The real-time RT-PCR was conducted to examine the expression of complement genes (*C1q, C2, C3, C4, CFI* and *CFD*) in the guinea pig model of long-term form deprivation-induced myopic retinal degeneration.

**Results:**

OCTA showed that retinal neuronal thickness and vascular density in superficial and deep layers of the macular zone as well as vascular density in the optic nerve head were progressively decreased from SM to PS and PS + CA (*p* < 0.05). The aqueous humour levels of C1q, C3, C3b/iC3b, C4, CFB, CFH, C2, C4b, C5 and CFI were significantly higher in high-myopic eyes compared to those in low-myopic eyes. Further subgroup analysis revealed the highest levels of complement components/fragments in the PS + CA group. The intraocular levels of complement factors particularly C3b/iC3b and C4 were negatively correlated with macular zone deep layer retinal thickness and vascular density and optic nerve head vascular density. The expression of *C2, C3* and *C4* genes was significantly higher in guinea pig eyes with myopic retinal degeneration compared to control eyes.

**Conclusions:**

The intraocular classical pathway and alternative pathway of the complement system are partially activated in pathological myopia. Their activation is related to the degeneration of retinal neurons and the vasculature in the macula and the vasculature in the optic nerve head.

## 1. Introduction

Myopia is an important public health problem around the world (Pascolini and Mariotti, 2012). It is estimated that approximately half of the world’s population will have myopia, of whom 10% will suffer from high myopia by 2050, especially in the east and southeast of Asia (Holden et al., 2016). The incidence of myopia to high myopia progression increased with age, especially after grade three and those with high myopia had a significantly increased lifetime risk of visual impairment compared with those with emmetropia (Tideman et al., 2016; Li et al., 2022). Myopia-mediated visual impairment was associated with axial length and the spherical equivalent (Jonas et al., 2021). Pathological myopia refers to a type of myopia accompanied by retinal/choroidal degenerative changes including posterior staphyloma, lacquer cracks, optic disc abnormalities, chorioretinal atrophy, choroidal neovascularisation and myopic maculopathy (Ohno-Matsui et al., 2016, 2021). Although pathological myopia often occurs in high myopia, it can also develop in low-to-moderate myopia (Ohno-Matsui et al., 2021). Currently, there is no medication to prevent or treat myopic retinal degeneration due to poor knowledge of the pathogenesis of the condition.

The neural retina is an extension of the brain and is connected to the brain via the optic nerve (Neuringer et al., 1988). Thus, the retina has many functional and anatomical features similar to those of the brain. Several degenerative diseases like Alzheimer’s disease and dementia have been reported to be attributable to chronic inflammation (Heneka et al., 2015; Pasqualetti et al., 2015). In retinal diseases, it is also reported that inflammation plays an important role in retinal neuronal degeneration with different causes, such as age-related macular degeneration (Kauppinen et al., 2016; Xu and Chen, 2022) diabetic retinopathy (Tang and Kern, 2011), and glaucomatous retinopathy (Baudouin et al., 2021). Microglia, the complement system and inflammasome activation are three key inflammatory pathways critically involved in retinal degeneration and have been targeted for therapy (Xu and Chen, 2016; Akhtar-Schäfer et al., 2018; Kaarniranta et al., 2018). Recent studies have uncovered strong links between altered immune response and myopia (Xue et al., 2021; Zeng et al., 2021). It has been shown that patients with inflammatory or autoimmune diseases have a higher incidence of myopia compared to those without (Herbort et al., 2011). Allergic conjunctivitis can promote myopic progression (Wei et al., 2018), and high-myopic eyes contain higher levels of inflammatory cytokines and complement proteins (Gao et al., 2015; Yuan et al., 2019; García-Gen et al., 2021). In addition, myopic refractive shifts were present in many ocular diseases such as multifocal chorioretinitis, punctate inner chorioretinopathy and diffuse subretinal fibrosis syndrome (Gross et al., 1993; Jampol, 1996; Nguyen and Foster, 1998). The results suggest that inflammation may play an important role in the development and progression of myopia and myopic retinopathy.

Among the three common retinal degeneration-related inflammatory pathways, complement system has been reported to be closely associated with myopia. Several altered systemic and local complement proteins or pathways were detected in chick and guinea pig models of myopia (Gao et al., 2015; Giummarra et al., 2018; Zeng et al., 2021). Patients with pathological myopia had higher intraocular levels of complement proteins (Xue et al., 2021). However, which complement pathways are activated in the progression of myopic retinopathy and exactly how complement activation promotes myopic progression remains unknown. In this clinical study, we investigated the levels of complement proteins involved in multiple complement pathways in the aqueous humour from myopic patients without and with different degrees of pathological myopia. We further investigated the relationship between intraocular complement proteins levels and myopia-related retinal neuronal and vascular degeneration. The upregulation of complement genes was further confirmed in the guinea pig model of myopia-induced retinal degeneration.

## 2. Materials and methods

### 2.1. Clinical samples

#### 2.1.1. Subjects

The clinical study was performed following the principles of the Declaration of Helsinki. Informed consent was obtained from all participants and the Institutional Review Board (IRB) at the Aier Eye Hospital Group approved the study (IRB number: AIER 2019IRB03). 147 myopic patients attending the Changsha Aier Eye Hospital, Hunan, China between November 2019 to October 2020 for Implantable Collamer Lens (ICLs) implantation were recruited for this study. The inclusion criteria were: (1) myopia; (2) receiving ICL surgery; (3) willing to participate in the study. Exclusion criteria were: (1) history of ocular trauma and ophthalmic surgery within 6 months; (2) history of retinal laser photocoagulation within 6 months; (3) use of antimetabolites, immunosuppressants or corticosteroids; (4) systemic inflammatory or autoimmune diseases; (5) pre-existing ocular inflammatory-related diseases. During data analysis, the participants were grouped into either high myopia (axial length ≧26.5 mm or spherical equivalent ≦-8.00D) and low myopia(axial length < 26.5 mm and spherical equivalent > −8.00D) (Tokoro, 1998), or with and without different degrees of pathological myopia (i.e., posterior staphyloma (PS) or with myopic chorioretinal atrophy (PS + CA).

Posterior staphyloma was diagnosed based on ocular B-scan ultrasonography. The diagnostic criteria were according to the definition of posterior staphyloma that an outpouching of a circumscribed posterior fundus region and had a curvature radius that was smaller than the curvature radius of the fellow eye (Spaide, 2014). Pathological myopia-related retinopathy was diagnosed using colour fundus images and scanning laser ophthalmoscope (SLO) examination. The diagnostic criteria were based on the META-PM classification and the definition of pathological myopia, including diffuse chorioretinal atrophy (myopic maculopathy category 2), patchy chorioretinal atrophy (myopic maculopathy category 3), macular atrophy (myopic maculopathy category 4), or presence of plus lesion, including neovascularization, Fuchs and lacquer cracks (Ohno-Matsui et al., 2015).

#### 2.1.2. Clinical examination

All subjects received comprehensive ophthalmic examinations including logMAR best corrected visual acuity (BCVA), slit lamp examination, intraocular pressure measurement (with a non-contact tonometer), axial length measurement (with an optical biometric device, IOL-Master700, Carl Zeiss, Meditec, Dublin, California), spherical equivalent (defined as a sphere plus a half negative cylinder measured by phoropter, NIDEK, ARK-1), ocular B-scan ultrasonography (Aviso, Quantel Medical, France), and fundus examination. Fundus examinations were recorded using RTVue XR Avanti Angio-OCT (OCTA, Optovue, Fremont, CA, USA) scanned in 6 mm × 6 mm in the macular zone (Supplementary Figures 1A–D) and 4.5 mm × 4.5 mm in the optic nerve head (Supplementary Figures 1E–G) and SLO (Optos, Daytona, Dunfermline, United Kingdom).

The below measurements were conducted in OCTA images using the AngioVue Software (Version 2018.1.1.63, Optovue, Inc.). Macular zone: (1) superficial retinal vessel density (SRVD); (2) deep layer retinal vessel density (DRVD); (3) superficial retinal thickness (SRT); (4) deep layer retinal thickness (DRT); (5) foveal vessel density (FD); (6) foveal avascular zone (FAZ); (7) FAZ perimeter (PERIM); (8) average thickness of ganglion cell complex (GCC) (Supplementary Figures 1A–D). Optic nerve head: (1) average thickness of retinal nerve fiber layers (average RNFL); (2) optic nerve head all vessel density (ONHVD-all vessels); (3) optic nerve head small vessel density (ONHVD -small vessels); (4) inside disc all vessel density (IDVD-all vessels); (5) inside disc small vessel density (IDVD - small vessels); (6) Peripapillary all vessel density (Peripapillary VD- all vessels); (7) Peripapillary small vessel density (Peripapillary VD - small vessels) (Supplementary Figures 1E–G).

#### 2.1.3. Aqueous sample collection

Aqueous humour was obtained immediately before ICL implantation. Approximately 100∼150 μL of aqueous humour was drawn from the anterior chamber through the limbus using a disposable sterile syringe. The sample was transferred into a sterile Eppendorf and stored at −80°C until measurement.

#### 2.1.4. Measurement of complement proteins

The levels of complement proteins in aqueous humour, including C1q, C3, C3b/iC3b, C4, CFB, CFH, C2, C4b, C5, C5a, CFD, MBL and CFI were assayed with the xMAP kits (No. HCMP1MAG-19K and HCMP2MAG-19K, Millipore, USA) using the Luminex XMAP system following manufacturer’s instructions (Luminex Technology, Austin, USA). 25 μL of aqueous humour from each sample (original fluid) was used in the measurement of C1q, C3, C3b/iC3b, C4, CFB and CFH. 12.5 μL of aqueous humour from each sample diluted with RD6-52 (1:1 dilution) was used to measure C2, C4b, C5, C5a, CFD, MBL and CFI.

### 2.2. Quantitative real-time PCR analysis of complement expression in form deprivation-induced myopic retinal degeneration

We previously developed a form deprivation-induced high myopia (FDM) in pigmented guinea pigs and the animals presented early signs of retinal neuronal degeneration after 15 weeks of form deprivation (Zeng et al., 2021). The study protocols were approved by the Animal Care and Ethics Committee of the Central South University (Ref: 2021SYDW0026) and all procedures were performed according to the Association for Research in Vision and Ophthalmology (ARVO) statement for the Use of Animals in Ophthalmic and Vision Research.

Using mRNA samples from this study (Zeng et al., 2021), we conducted qRT-PCR analysis on complement related genes, including *C1q, C2, C3, C4(C4a), CFD*, and *CFI*. The mRNA from three groups of retinae was used in this study: FDM retinae, self-control retinae, and retinae from animals without form deprivation. Seven retinas were included in each group. 400 ng RNA from each retina sample was used to synthesize cDNA using a Reverse Transcription kit (Vazyme, Nanjing, China). The cDNA was diluted to 1.25 ng/μl for the subsequent qRT-PCR. qTR-PCR was conducted in 96-well plates using a lightcycle96@Real-Time PCR System (Roche, Germany) and each sample was triplicated. The primers were purchased from Tsingke Biotechnology (Beijing, China) and detailed in Supplementary Table 8. *Actb* was used as a house-keeping gene. Each reaction (10 μL of volume) contained 1 μL of primer (10 μM), 2 μL cDNA, 5 μL Maxima SYBR Green Master Mix (2X) (Cat. K0252, Thermo Fisher Scientific, MA, USA) and 2 μL ddH2O. The reaction was conducted by 45 cycles of 95°C for 10 s, 60°C for 15 s and 72°C for 10 s (single acquire). The relative expression of candidate genes was obtained using the comparative threshold cycle (2^–Δ^ ^Δ^ ^Ct^) method (Livak and Schmittgen, 2001).

## 3. Statistics

Data analyses were performed using the Statistical Package for Social Sciences software (SPSS, V. 21.0, IBM Corp., USA). The Kolmogorov-Smirnov test was used to determine the clinical study data normality. The continuous variables with homogeneity were compared by one-way Analysis of Variance (ANOVA), followed by the Bonferroni’ *post hoc* analysis. For those variables that did not present homogeneity of variances, the Kruskal-Wallis test was used. Unadjusted linear regression was employed to examine the coefficients of two dependent variables. Chi-square analysis was used to analyze categorical variables such as gender, family history of myopia and history of photocoagulation etc. All continuous values were expressed as mean ± SD or mean ± SEM, and the categorical values were expressed as *N* (%). *P* < 0.05 was considered statically significant.

## 4. Results

### 4.1. Clinical study

#### 4.1.1. Clinical characteristics of the participants

Of the 147 participants, 60 had low-myopia, 87 had high-myopia. There was no significant difference in age, gender, BCVA and family history of myopia between the two groups. As expected, the spherical equivalent and axial length were significantly different between the two groups (Supplementary Table 1).

#### 4.1.2. Complement proteins levels in the aqueous humour in eyes with different degrees of myopia

We measured 13 complement components/fragments, four (C1q, C2, C4, and C4b) in the classical pathway (CP), four (CFB, CFH, CFI, and CFD) in the alternative pathway (AP), one (MBL) in the lectin pathway and four (C3, C3b/iC3b, C5 and C5a) in the common pathway. C5a was detected only in 4 out of 147 samples (ranging from 1.59∼12.44 pg/mL, sensitivity level: 1.14 pg/mL). Therefore, we did not conduct any further analysis on C5a. C5 was below the detectable limit (0.68 ng/mL) in 26 out of 147 samples, and 20 out of the 26 undetectable samples were in the low myopia group. In addition, the level of C2 was below the detectable threshold (0.25 ng/mL) in six samples of the low myopia group. Other complement components and complement fragments were detected in all samples.

When comparing the aqueous humour complement levels between the two groups, all components of the CP (C1q, C2, C4 and C4b) and the common pathway (C3, C3b/iC3b and C5) were significantly higher in the high-myopia group. In the AP, the aqueous levels of CFB and CFI were significantly higher in high myopic eyes. The levels of CFH, CFD and MBL did not differ between the two groups (Table 1).

**TABLE 1:** Complement proteins levels in the aqueous humor of patients with high myopia and low myopia (ng/ml).

	Low myopia	High myopia	*P*-values*
	**(*n* = 60)**	**(*n* = 87)**	
**Complement components/fragments of the CP**			
C1q	6.21 ± 3.32	9.31 ± 6.58	<0.001
C2	0.93 ± 0.52	2.15 ± 1.65	<0.001
C4	151.60 ± 55.00	197.67 ± 78.52	<0.001
C4b	18.59 ± 13.26	33.01 ± 27.21	<0.001
**Complement components/fragments of the AP**			
CFB	126.28 ± 35.65	142.57 ± 38.54	0.01
CFH	39.40 ± 17.53	42.11 ± 21.92	0.43
CFI	63.38 ± 20.84	76.12 ± 35.13	0.007
CFD	33.03 ± 9.77	32.39 ± 10.55	0.71
**Lectin pathway**			
MBL	0.18 ± 0.14	0.19 ± 0.14	0.47
**Complement components/fragments of the shared pathway**			
C3	112.02 ± 30.46	139.48 ± 49.28	<0.001
C3b/iC3b	73.44 ± 39.64	110.74 ± 85.33	<0.001
C5	1.77 ± 1.53	3.85 ± 2.81	<0.001

*Student’s t-test.

#### 4.1.3. Complement proteins levels in the aqueous humour in eyes with different degrees of pathological myopia

To understand if the complement system is involved in pathological myopia, we classified the subjects into three groups based on the severity of retinal degeneration: simple myopia (SM, without pathological myopia, *n* = 78), posterior staphyloma (PS, *n* = 39) and posterior staphyloma with myopic chororetinal atrophy (PS + CA, *n* = 30), including 27 diffuse chorioretinal atrophy, 3 patchy chorioretinal atrophy (Supplementary Figure 2). Apart from the spherical equivalent and axial length, there was no difference in age, gender, and history of myopia between the three groups (Supplementary Table 2).

One-way ANOVA analysis showed that the aqueous levels of the complement components and fragments in the CP (C1q, C4 and C4b), AP (CFB, CFH and CFI) and the common pathway (C3, C3b/iC3b, and C5) significantly differed among the three groups (Table 2). Further *post hoc* analysis uncovered the largest difference lies in SM vs PS + CA. The aqueous levels of these complement proteins progressively increase from SM to PS, with the highest values in the PS + CA group (Table 2). We did not detect any statistical difference in the aqueous levels of CFD and MBL among the three groups (Table 2).

**TABLE 2:** Complement proteins levels in the aqueous humor from different degrees of pathological myopia patients (ng/ml).

	SM (*n* = 78)	PS (*n* = 39)	PS + CA (*n* = 30)	*P*-value (all)^a^	*P*-value^b^ SM vs PS	*P*-values^b^ SM vs PS + CA	*P*-values^b^ PS vs PS + CA
**Complement components/fragments of the CP**							
C1q	6.44 ± 3.37	7.92 ± 3.85	12.37 ± 9.31	<0.001	0.04	0.002	0.02
C2	1.22 ± 0.79	1.52 ± 1.04	2.93 ± 2.29	<0.001	0.12	<0.001	0.003
C4	157.10 ± 60.04	186.64 ± 76.09	225.33 ± 79.32	<0.001	0.02	<0.001	0.04
C4b	22.17 ± 18.40	24.83 ± 22.29	42.99 ± 30.41	<0.001	0.49	0.001	0.006
**Complement components/fragments of the AP**							
CFB	124.79 ± 35.27	140.60 ± 33.77	158.80 ± 40.18	<0.001	0.02	<0.001	0.04
CFH	36.96 ± 14.41	40.38 ± 17.80	52.32 ± 30.24	0.002	0.27	0.01	0.06
CFI	66.62 ± 25.54	63.14 ± 24.53	92.23 ± 40.26	<0.001	0.48	0.002	0.001
CFD	31.83 ± 9.73	31.92 ± 10.04	35.72 ± 11.36	0.18	0.97	0.08	0.15
**Lectin pathway**							
MBL	0.17 ± 0.13	0.19 ± 0.12	0.23 ± 0.18	0.14	0.31	0.11	0.38
**Complement components/fragments of the shared pathway**							
C3	121.65 ± 40.59	131.16 ± 49.44	141.75 ± 46.03	0.1	0.27	0.03	0.37
C3b/iC3b	75.67 ± 44.19	89.62 ± 51.88	154.77 ± 114.20	<0.001	0.13	<0.001	0.006
C5	2.15 ± 1.80	3.03 ± 2.08	5.18 ± 3.52	<0.001	0.02	<0.001	0.005

SM, simple myopia; PS, posterior staphyloma; CA, myopic chorioretinal atrophy; PS, posterior staphyloma; PS + CA, posterior staphyloma with myopic chorioretinal atrophy.

^a^ANOVA.

^b^Bonferroni’post hoc analysis.

#### 4.1.4. Retinal neuronal and vascular degeneration in different groups of pathological myopia

The pathological myopic-related changes in retinal neurons and the vasculature around the optic nerve head and the macular zone were examined by angio-OCT (OCTA) (Supplementary Figure 1). In the macular zone, there was a significant difference among the three groups in SRVD, DRVD, SRT, DRT, and FD, but not FAZ, PERIM and average GCC. Further analysis of the subgroups showed that retinal vascular densities (SRVD, DRVD and FD) and neuronal thickness (SRT and DRT) were significantly reduced in the PS + CA group compared to SM and PS groups. A trend of reduction in DRVD, SRT, and DRT was also observed in the PS group compared to SM (Supplementary Table 3). These results suggest a progressive loss of macular zone blood vessels and neurons from SM to PS and PS + CA.

In the optic nerve head, the average RNFL showed no significant difference between the groups. The vessel densities, including all vessels and small vessels, were progressively reduced from PS to PS + CA, particularly the peripapillary vessel densities. Interestingly, the small vessel density inside the disc (IDVD-small vessels) was higher in PS than in SM (Supplementary Table 3). Our results suggest peripapillary vascular degeneration in myopic retinopathy.

#### 4.1.5. The relationship between intraocular complement components/fragments and retinal neuronal and vascular degeneration

To understand if intraocular complement levels are related to retinal neuronal and vascular degeneration, we further analyzed the correlation between the laboratory and clinical parameters. Weak, but statistically significant negative correlations were observed between intraocular complement proteins (C4 and C3b/iC3b) and superficial retinal thickness and superficial vascular density (Supplementary Tables 4, 5). Whereas strong negative correlations were observed between intraocular complement proteins and the macular zone DRT and DRVD (Tables 3, 4; Supplementary Figure 3).

**TABLE 3:** The correlations between intraocular complement levels and DRT in the macular zone.

	All group (*n* = 147)		SM (*n* = 78)		PS (*n* = 39)		PS + CA (*n* = 30)	
	**β (SE)**	***P*-values^†^**	**β (SE)**	***P*-values^†^**	**β (SE)**	***P*-values^†^**	**β (SE)**	***P*-values^†^**
**Complement components/fragments of the CP**								
C1q	–0.17 (0.03)	<0.001	–0.03 (0.04)	0.46	–0.09 (0.04)	0.02	–0.56 (0.14)	<0.001
C2	–0.04 (0.01)	<0.001	–0.00 (0.01)	0.74	–0.01 (0.01)	0.28	–0.09 (0.04)	0.06
C4	–1.60 (0.45)	<0.001	0.11 (0.67)	0.87	–1.23 (0.79)	0.13	–3.58 (1.43)	0.02
C4b	–0.51 (0.15)	0.001	–0.06 (0.21)	0.76	–0.35 (0.24)	0.16	–0.90 (0.60)	0.14
**Complement components/fragments of the AP**								
CFB	–0.60 (0.23)	0.01	0.76 (0.36)	0.04	–0.35 (0.34)	0.32	–2.81 (0.54)	<0.001
CFH	–0.34 (0.11)	0.002	0.18 (0.16)	0.27	–0.38 (0.19)	0.052	–1.18 (0.33)	0.002
CFI	–0.54 (0.19)	0.007	0.28 (0.27)	0.31	–0.14 (0.28)	0.61	–2.38 (0.70)	0.003
CFD	–0.07 (0.06)	0.25	0.14 (0.11)	0.2	–0.04 (0.11)	0.74	–0.58 (0.16)	0.001
**Lectin Pathway**								
MBL	0.00 (0.00)	0.77	0.00 (0.00)	0.28	0.00 (0.00)	0.46	–0.00 (0.00)	0.11
**Complement components/fragments of the shared pathway**								
C3	–0.80 (0.27)	0.003	0.09 (0.44)	0.84	–1.28 (0.47)	0.01	–2.17 (0.69)	0.005
C3b/iC3b	–1.95 (0.42)	<0.001	0.45 (0.46)	0.33	–1.16 (0.55)	0.04	–6.78 (1.69)	<0.001
C5	–0.04 (0.01)	0.003	–0.03 (0.02)	0.13	0.00 (0.02)	0.85	–0.04 (0.05)	0.48

β, regression coefficient; SE, standard error; SM, simple myopia; PS, posterior staphyloma; CA, myopic chorioretinal atrophy; PS, posterior staphyloma; PS + CA, posterior staphyloma with myopic chorioretinal atrophy; DRT, deep retinal layer thickness.

^†^Unadjusted Linear Regression.

**TABLE 4:** The correlation between intraocular complement levels and DRVD in the macular zone.

	Group (*n* = 147)		SM (*n* = 78)		PS (*n* = 39)		PS + CA (*n* = 30)	
	**β (SE)**	***P*-values**	**β (SE)**	***P*-values^†^**	**β (SE)**	***P*-values^†^**	**β (SE)**	***P*-values^†^**
**Complement components/fragments of the CP**								
C1q	–0.46 (0.08)	<0.001	–0.03 (0.09)	0.77	–0.44 (0.11)	<0.001	–0.72 (0.26)	0.01
C2	–0.11 (0.02)	<0.001	0.02 (0.02)	0.33	–0.09 (0.03)	0.01	–0.16 (0.07)	0.04
C4	–4.73 (1.11)	<0.001	–1.68 (1.64)	0.31	–3.96 (2.49)	0.12	–4.88 (2.37)	0.052
C4b	–1.08 (0.38)	0.005	–0.03 (0.53)	0.95	–0.95 (0.77)	0.23	–1.00 (0.98)	0.32
**Complement components/fragments of the AP**								
CFB	–2.20 (0.57)	<0.001	–0.32 (0.92)	0.73	–2.53 (1.00)	0.02	–1.89 (1.24)	0.14
CFH	–0.83 (0.27)	0.003	0.13 (0.39)	0.75	–1.23 (0.58)	0.04	–1.05 (0.63)	0.11
CFI	–1.56 (0.48)	0.002	0.08 (0.67)	0.9	–1.78 (0.81)	0.04	–1.94 (1.32)	0.16
CFD	–0.27 (0.16)	0.1	0.25 (0.27)	0.35	–0.38 (0.35)	0.29	–0.63 (0.30)	0.045
**Lectin Pathway**								
MBL	–0.00 (0.00)	0.21	–0.00 (0.00)	0.92	–0.00 (0.00)	0.43	–0.00 (0.00)	0.37
**Complement components/fragments of the shared pathway**								
C3	–0.88 (0.69)	0.21	0.97 (1.10)	0.38	–2.97 (1.56)	0.07	–0.52 (1.33)	0.7
C3b/iC3b	–4.89 (1.06)	<0.001	0.63 (1.14)	0.58	–4.17 (1.68)	0.02	–7.54 (3.19)	0.03
C5	–0.14 (0.03)	<0.001	–0.04 (0.04)	0.38	–0.10 (0.07)	0.19	–0.12 (0.08)	0.14

β, regression coefficient; SE, standard error; SM, simple myopia; PS, posterior staphyloma; CA, myopic chorioretinal atrophy; PS, posterior staphyloma; PS + CA, posterior staphyloma with myopic chorioretinal atrophy; DRVD, deep layer retinal vessel density.

^†^Unadjusted Linear Regression.

When all participants were analyzed together, apart from CFD and MBL, all other complement components/fragments showed a strong inverse correlation with macular zone DRT (Table 3); whereas except for C3, CFD and MBL, other components/fragments showed a strong correlation with macular zone DRVD (Table 4) with the highest being C3b/iC3b followed by C4 (β = −1.95 and −1.60 for DRT and −4.89 and −4.73 for DRVD respectively, *P* < 0.001, Tables 3, 4; Supplementary Figure 3). Further subgroup analysis revealed that most of the DRT-related correlations were in the PS + CA group (Table 3), whereas the DRVD-related correlations were more often in the PS group than the PS + CA group (Table 4).

In the optic nerve head, weak correlations were observed between RNFL and complement parameters such as C1q, CFB, CFH, C3, C3b/iC3b and MBL (with low values of regression coefficient) (Supplementary Table 6). Regarding the parameter of peripapillary VD-all vessels, significant reverse correlations with C1q, C2, C4, CFB, CFI, C3b/iC3b and C5 were detected with the highest correlation in C3b/iC3b, followed by C4 and CFB (β = −5.72, −3.37 and −2.90 for C3b/iC3b, C4 and CFB respectively, *P* < 0.001, Supplementary Table 7 and Supplementary Figure 4). Subgroup analysis showed that the negative correlations between peripapillary VD-all vessels and complement proteins were predominately in the PS + CA group.

Taken together, our results suggest that intraocular activation of the CP and AP of the complement system is related to macular zone deep layer retinal neuronal and vascular degeneration as well as the peripapillary vascular degeneration in pathological myopia.

#### 4.1.6. Complement gene expression in myopic retinal degeneration of guinea pigs

**FIGURE 1:**
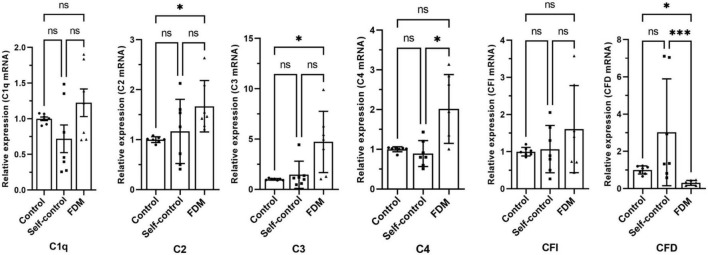
The relative gene expression of complement factors/components in different groups of guinea pig retina by qRT-PCR. Data presented as fold change of gene expression compared to control groups. Mean ± SEM, *n* = 7/group. ns, no significant difference, *p < 0.05. ***p < 0.001. One-way ANOVA followed by Dunn’s Multiple comparison test. FDM, form-deprivation induced myopia.

Real-time RT-PCR showed that the expression of *C2, C3* and *C4(C4a)* in the FDM retina was significantly higher compared to that in self-controls or in eyes from control guinea pigs. Interestingly, the expression of *CFD* was reduced in FDM eyes compared to the controls (Figure 1). No significant difference was observed in *C1q* and *CFI* genes (Figure 1).

## 5. Discussion

In this study, we detected significantly higher intraocular levels of complement proteins involved in the classic pathway (C1q, C2, C4 and C4b) and alternative pathway (CFB, CFI, C3b/iC3b) in human eyes with high-myopia or with pathological myopia. We further found that higher intraocular levels of C4 and C3b/iC3b were negatively correlated with the deep layer retinal thickness and microvascular density in the macula, as well as with the optic nerve head vascular density. Higher levels of retinal complement expression were also detected in a guinea pig model of myopia-induced retinal degeneration (Zeng et al., 2021). Complement fragments C4b, C3b/iC3b and C5a are evidence of complement activation. We detected C4b and C3b/iC3b but not C5a in the aqueous humor of myopic eyes, indicating partial activation of the intraocular complement system. Our results suggest that partial activation of the intraocular complement system is related to myopic retinopathy, particularly the degeneration of retinal neurons in the macula and microvascular degeneration in both the macula and optic disc.

Previous studies have reported the upregulation of the complement pathway, including C1q, C1qa, C2 and C1qb during the development stages of myopia in guinea pigs and chicks (Riddell and Crewther, 2017; Zeng et al., 2021). These animal models of myopia are valuable tools for understanding the mechanism of myopia onset and progression. All participants in our study are myopic, so the data are not suitable for evaluating the role of the complement system in myopia onset. However, by classifying the participants into low-myopia and high-myopia groups and into myopia with or without retinopathy, we could interrogate the involvement of intraocular complement system in myopia progression and the development of myopic retinopathy. The levels of C1q, C2, C4, C4b, CFB, CFI, C3, C3b/iC3b, and C5 in the aqueous humour were significantly higher in high-myopic eyes compared to those in low-myopic eyes. MBL was detected in all samples (albeit at low levels), but there was no difference between the two groups. Our results suggest that partial activation of the intraocular CP and AP of the complement system is involved in the progression from low-myopia to high-myopia.

Proteins in the intraocular fluids (aqueous humour and vitreous body) originate predominately from cells inside the eye when the blood retinal barrier is intact. In the context of complement proteins, retinal cells including neurons, glial cells and retinal pigment epithelial cells constitutively express complement and complement regulatory genes and their expression can be upregulated under oxidative stress and inflammatory conditions (Anderson et al., 2010; Luo et al., 2013; Xu and Chen, 2016; Liu et al., 2020). The alterations in the composition and quantity of intraocular proteins can truthfully reflect the health condition of intraocular tissues, particularly the retina. To understand the role of the complement system in pathological myopia, we classified the participants into three groups based on the degree of pathological myopia, SM (no pathological myopia), myopia-related PS (without pathological myopia-related retinopathy) and PS + CA (with myopic chorioretinal atrophy). This study design allowed us to investigate the link between myopia-mediated retinal neuronal and vascular changes and intraocular complement activation. Indeed, we found progressive thinning of the macular zone retinal neurons and the gradual loss of microvasculature in the macula (both superficial and deep layers) and the optic nerve head during the progression of pathological myopia. There was no significant difference in the vascular density inside the optic disc in different groups. Reduced retinal microvascular density has been observed in high-myopic eyes (Li et al., 2017; Ucak et al., 2020; Shi et al., 2021; Moon et al., 2023), but not in high-myopic anisometropia eyes (Wang et al., 2022), although the superficial and deep layer retinal neurons were thinner in high-myopic eyes including high-myopic anisometropia eyes compared to contralateral control eyes (Ucak et al., 2020; Shi et al., 2021; Wang et al., 2022). Our results were in line with previous reports.

We found that the levels of intraocular complement proteins, including C1q, C2, C4, C4b, CFB and C3b/iC3b progressively increase from SM to PS and PS + CA. More importantly, we observed negative correlations between intraocular complement levels and macular zone deep layer retinal thickness and microvascular density, particularly in the severe pathologcial myopia group (i.e., PS + CA). Among different complement proteins, the strongest correlation was observed in C4, followed by C3b/iC3b and CFB, suggesting that the CP complement system (along with AP) may be critically involved in retinal neuronal and vascular degeneration, particularly in the outer retinal layer, in pathological myopia-related retinopathy.

It is important to note that, although a significant amount of C3 (range: 48.80∼290.04 ng/ml) and its fragment C3b/iC3b (range: 12.17∼455.43 ng/ml) in this clinical study was detected in all samples, the aqueous level of C5 was very low (range: 0∼16.97 ng/ml) and C5a was only detected in 4 out of 147 myopic eyes. The results indicate that the intraocular complement cascade is only activated to the C3 cleavage step in the myopic eyes. The C3-derived complement fragments C3a and C3b are known to be involved in various immune responses. C3a can induce chemotaxis, immune cell activation, angiogenesis, and macrophage-to-myofibroblast transition through its receptor C3aR (Sacks, 2010; Wang et al., 2022); whereas C3b can opsonize pathogens and apoptotic cells for phagocytosis, as well as form the C3 convertase with CFB (C3bBb) (Xu and Chen, 2016). In our study, the level of intraocular C3b/iC3b increased progressively from simple myopia (SM: 75.67 ± 44.19 ng/ml) to myopia with posterior staphyloma (PS: 89.62 ± 51.88 ng/ml), and to myopia with posterior staphyloma and chorioretinal atrophy (PS + CA: 154.77 ± 114.20 ng/ml) (Table 2). The level of C3b/iC3b in the PS + CA myopic eye is at the same range as the level of C3 (141.75 ± 46.03 ng/ml, Table 2). This suggests that the level of intraocular complement activation (although partially) is in line with the severity of myopic retinopathy. The intraocular complement system may participate in myopic retinal degeneration by modulating retinal immune response through the release of C3a and C3b although further functional studies will be needed to confirm this.

This study has several limitations. Firstly, we do not have an emmetropic control group due to the unavailability of aqueous humour samples. Secondly, clinical evaluation and aqueous humour samples collection were conducted at the same time, and we did not follow up the participants during disease progression, therefore, it is difficult to establish the causal link between intraocular complement activation and myopic retinal degeneration. Myopia-induced retinal neuronal and vascular degeneration is a slow process. If intraocular complement activation is one of the key drivers, complement activation would exist many years prior to retinal degeneration. Third, we did not measure the circulating levels of complement protein and complement activity. We do not know if higher intraocular complement levels are due to systemic complement activation in the participants. Fourth, among different degrees of pathological myopia, only PS and PS + CA were included in the study. Further studies will be needed to understand the role of complement activation in other types of myopic retinopathy including the high prevalence peripheral retinal degeneration (Lam et al., 2005) and the more severe myopic maculopathy such as myopic choroidal neovascularization and macular hemorrhage. Finally, we were not able to conduct functional study to test the role of the complement system in the animal model of myopia-induced retinal degeneration due to the lack of guinea pig-specific complement inhibitors.

## 6. Conclusion

In this study, we uncovered progressive partial intraocular complement activation during the progression of low-myopic to high-myopia. Importantly, we found that the intraocular classical pathway and alternative pathway of the complement system are involved in the development and progression of pathological myopia, in particular, the outer retinal neuronal and vascular degeneration and optic head vascular degeneration.

## Data availability statement

The raw data supporting the conclusions of this article will be made available by the authors, without undue reservation.

## Ethics statement

All procedures concerning the collection of aqueous humour were performed following the principles of the Declaration of Helsinki. Informed consent was obtained from all participants and the Institutional Review Board (IRB) at the Aier Eye Hospital Group approved the study (IRB number: AIER 2019IRB03). The animal study protocols were approved by the Animal Care and Ethics Committee of the Central South University (Ref: 2021SYDW0026) and all procedures were performed according to the Association for Research in Vision and Ophthalmology (ARVO) statement for the Use of Animals in Ophthalmic and Vision Research.

## Author contributions

LZ, HX, and ZY concepted and designed study, revised the manuscript. LZ, XL, YT, MW, JW, and LH collected experimental data. DL, WC, and RZ collected aqueous humour. LZ, WP, HX, and ZY analyzed data. LZ and HX drafted the manuscript. All authors contributed to the article and approved the submitted version.
