# Supplementary material for: Intraocular complement activation is related to retinal vascular and neuronal degeneration in myopic retinopathy
Source: Front Cell Neurosci. 2023 Jun 28;17:1187400. doi: 10.3389/fncel.2023.1187400 (PMC10336352; doi:10.3389/fncel.2023.1187400)
Supplement: Supplementary file 1 [file Data_Sheet_1.docx]

Supplementary Material

Intraocular Complement Activation Is Related to Retinal Vascular and Neuronal Degeneration in Myopic Retinopathy

Ling Zeng^[[1]](#footnote-1),2,3^, Xiaoning Li^4,2^, Wei Pan^3^, Yao Tang^3,2^, Ding Lin^2^, Min Wang^5^, Wang Cai^2^, Ruiling Zhu^2^, Jianbo Wan^2^, Linghua Huang^2^, Heping Xu^1,3,6*^, Zhikuan Yang^2,1,3*^

Aier School of Ophthalmology, Central South University, Changsha, China.

^2^ Changsha Aier Eye Hospital, Changsha, Hunan, China.

^3^ Aier Institute of Optometry and Vision Science, Aier Eye Hospital Group, Changsha, China.

^4^ Aier School of Optometry and Vision Science, Hubei University of Science and Technology, Xianning, Hubei, China.

^5^ Shanghai Aier Eye Hospital, Shanghai, China.

^6^ The Wellcome-Wolfson Institute for Experimental Medicine, School of Medicine, Dentistry and Biomedical Sciences, Queen’s University Belfast, 97 Lisburn Road, Belfast, BT9 7BL, UK.

*** Correspondence:**

Corresponding to Professor Heping Xu, Tel: +44(0)28909 76463, Email: [heping.xu@qub.ac.uk](mailto:heping.xu@qub.ac.uk) or Professor Zhikuan Yang, +86(0)13380071988, Email: [yangzhikuan@aierchina.com](mailto:yangzhikuan@aierchina.com)

1. **Supplementary Figures**


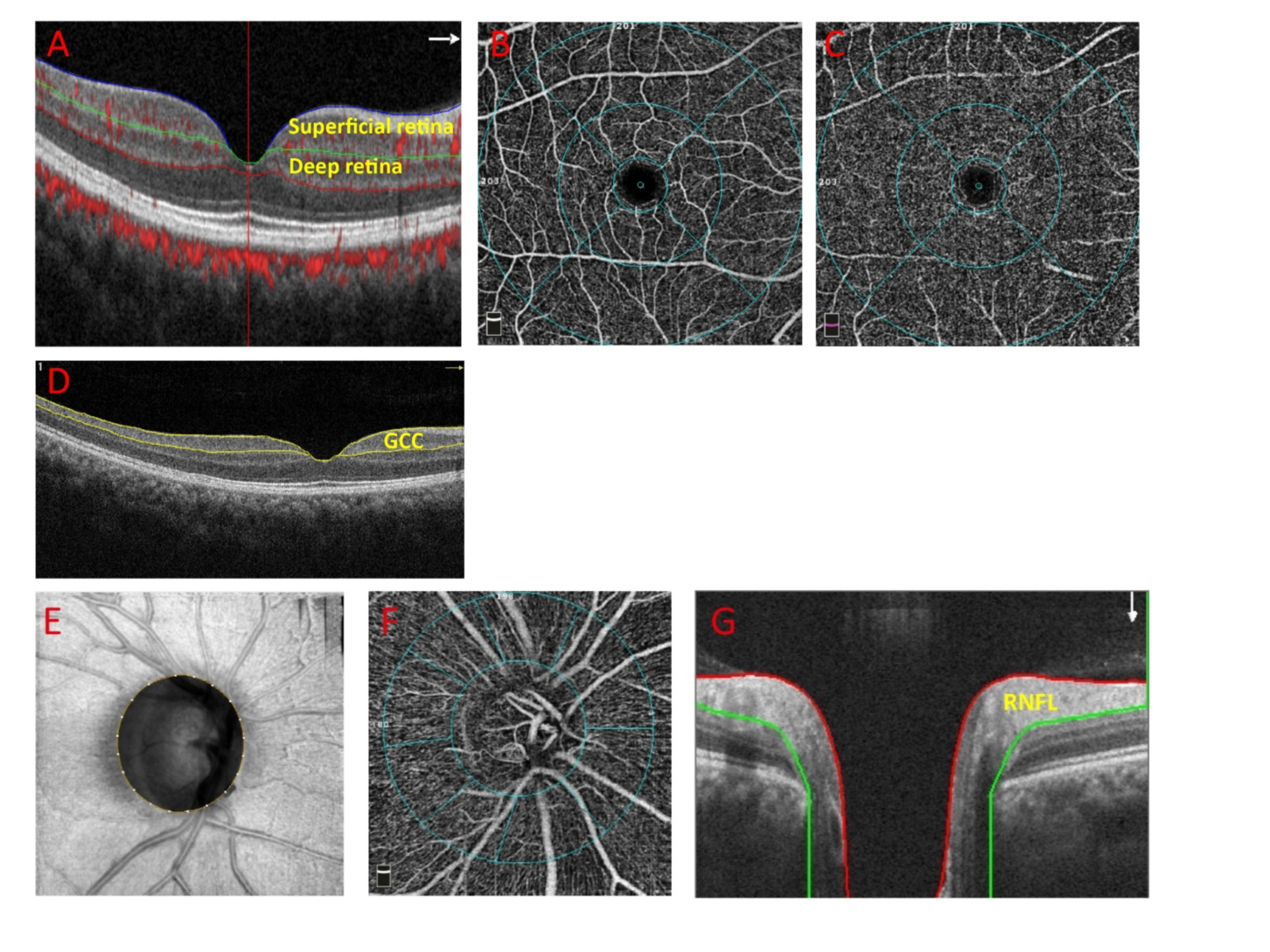


**Supplementary Figure S1.** Representative OCTA images showing the measurement of retinal neurons and the vasculature in the macular zone and around the optic nerve head. (A) Cross section of OCTA image showing different retinal layers of the macular zone (6x6 mm rectangle scan). The superficial and deep retinal layers were automatically marked using the AngioVue system (Version 2018.1.1.63, Optovue, Inc.); (B) Superficial retinal vessels; (C) Deep retinal vessels; (D) Ganglion cell complex (GCC) including nerve fiber layer (NFL), and ganglion cell layer (GCL) and inner plexiform layer (IPL). (E-G) Representative OCTA image of the optic disc head. (E) optic disc head; (F) Radial peripapillary capillaries (RPC). The inside optic area is the area surround by the inner blue circle; the peripapillary region area is between the two blue circle. (G) Retinal nerve fiber layer (RNFL) in the optic nerve head.

**
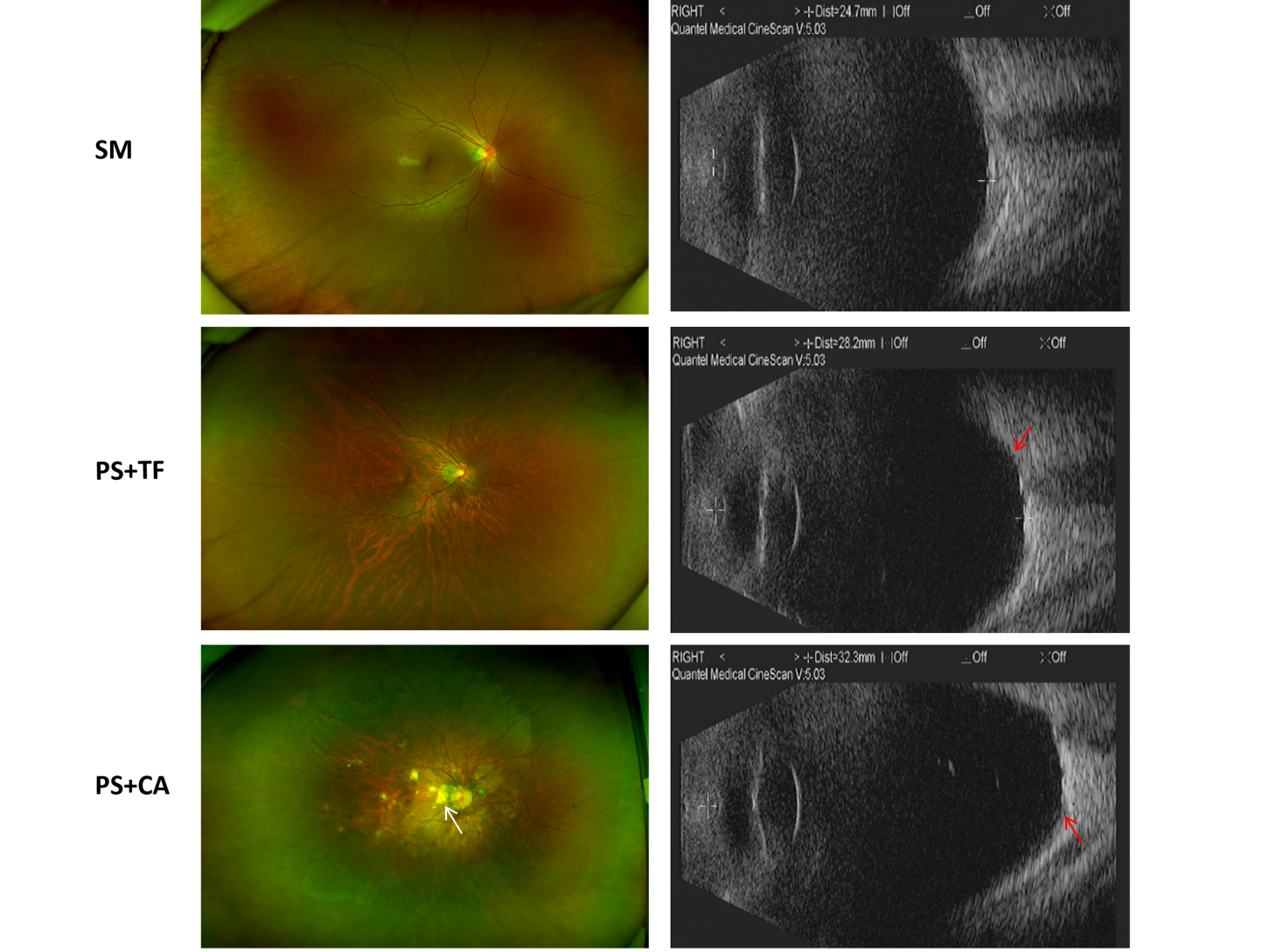
**

**SM**

**PS**

**PS+CA**

**Supplementary Figure S2.** Representative fundus images and B-scan ultrasonography images of different groups of myopia. SM: simple myopia; PS: posterior staphyloma; CA: myopic chorioretinal atrophy; PS: posterior staphyloma; PS+CA: posterior staphyloma with myopic chorioretinal atrophy. White arrow indicates diffuse chorioretinal atrophy. Red arrow indicates posterior staphyloma.

**
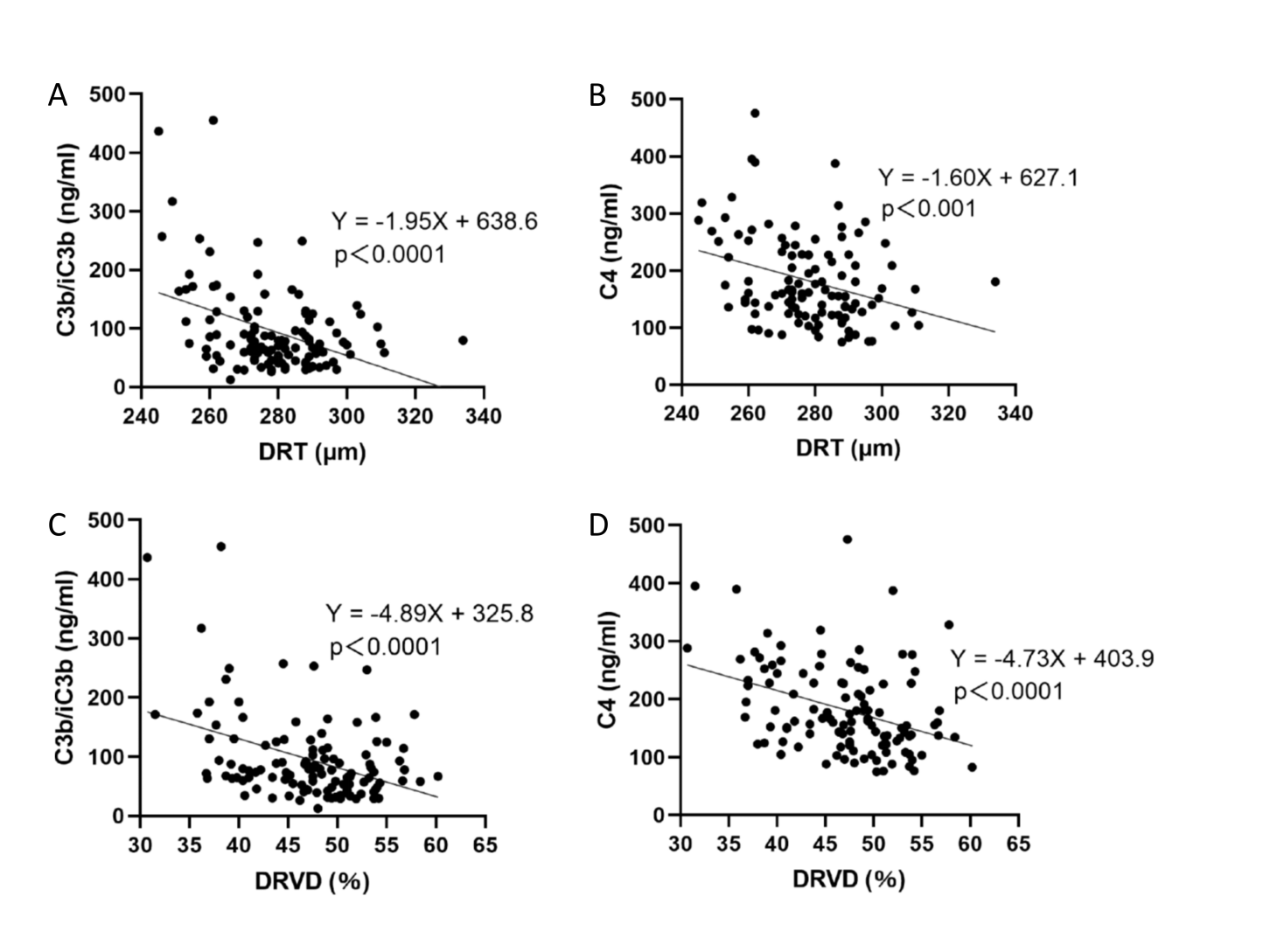
**

**Supplementary Figure S3.** The correlations between complement components/fragments (C3b/iC3b and C4) and the macular zone deep retinal thickness and deep retinal vessel density. (A) the correlation between C3b/iC3b and DRT; β=-1.95, *p* < 0.0001; N = 114. (B) the correlation between C4 and DRT; β = - 1.60, *p* < 0.001; N = 114. (C) the correlation between C3b/iC3b and DRVD; β = - 4.89, *p* < 0.0001; N = 114. (D) the correlation between C4 and DRVD; β = - 4.73, *p* < 0.0001; N = 114. Statistical methods: Unadjusted Linear Regression; DRT: deep retinal thickness, DRVD: deep retinal vessel density.


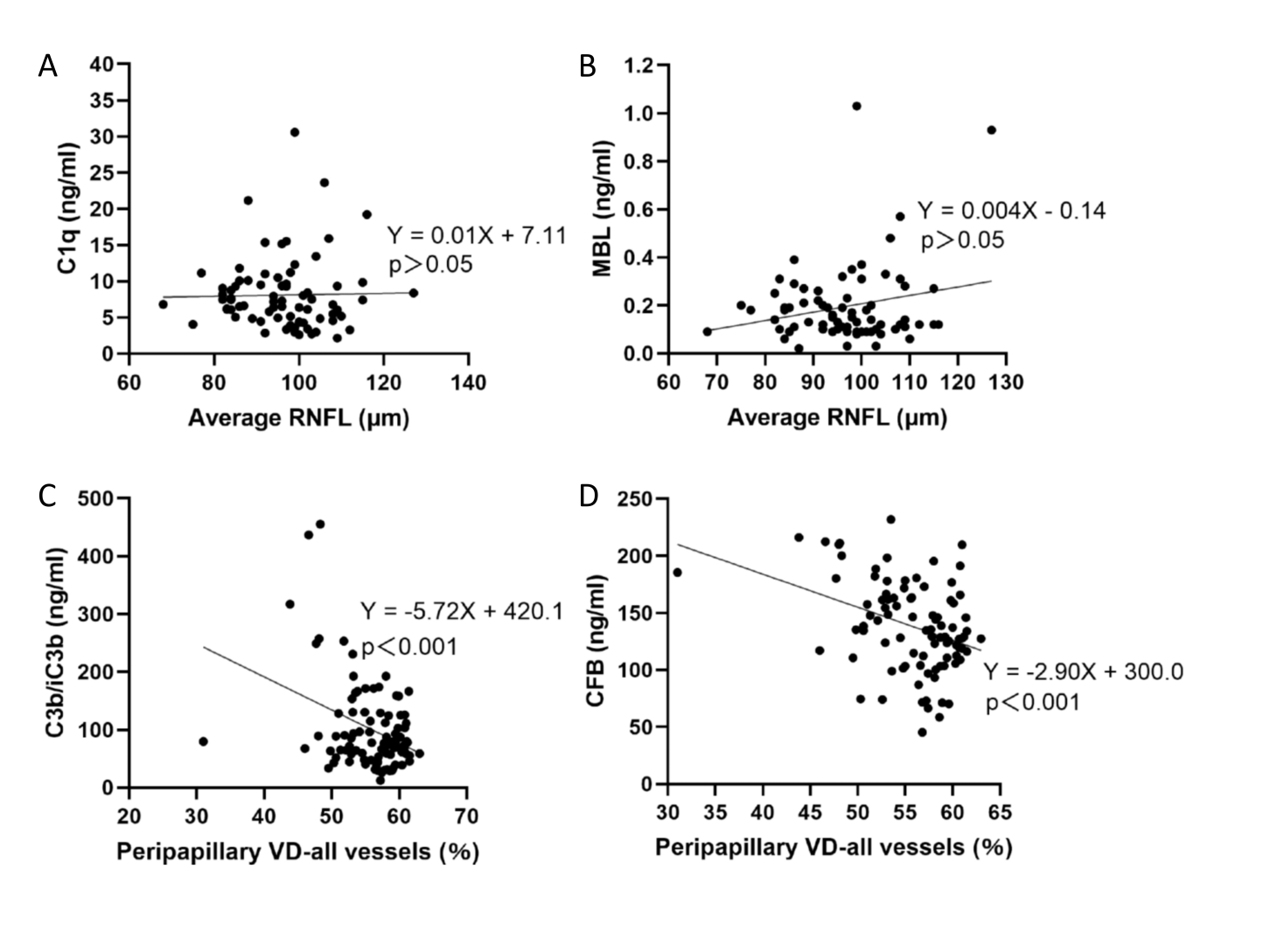


**Supplementary Figure S4.** The correlations between complement components/fragments (C1q, MBL, C3b/iC3b and CFB) and the optic nerve head average RNFL and peripapillary VD-all vessels. (A) the correlation between C1q and average RNFL; β = 0.01, *p >* 0.05; N = 77. (B) the correlation between MBL and average RNFL; β = 0.004, *p >* 0.05; N = 77. (C) the correlation between C3b/iC3b and peripapillary VD-all vessels; β = -5.72, *p* < 0.001; N = 94. (D) the correlation between CFB and peripapillary VD-all vessels; β = - 2.90, *p* < 0.001; N = 94. Statistical methods: Unadjusted Linear Regression; average RNFL: average retinal nerve fiber layers; peripapillary VD-all vessels: Peripapillary all vessel density.

1. [↑](#footnote-ref-1)
