# Supplementary material for: Intraocular complement activation is related to retinal vascular and neuronal degeneration in myopic retinopathy
Source: Front Cell Neurosci. 2023 Jun 28;17:1187400. doi: 10.3389/fncel.2023.1187400 (PMC10336352; doi:10.3389/fncel.2023.1187400)
Supplement: Supplementary file 2 [file Data_Sheet_2.docx]

Supplementary Material

Intraocular Complement Activation Is Related to Retinal Vascular and Neuronal Degeneration in Myopic Retinopathy

Ling Zeng^[[1]](#footnote-1),2,3^, Xiaoning Li^4,2^, Wei Pan^3^, Yao Tang^3,2^, Ding Lin^2^, Min Wang^5^, Wang Cai^2^, Ruiling Zhu^2^, Jianbo Wan^2^, Linghua Huang^2^, Heping Xu^1,3,6*^, Zhikuan Yang^2,1,3*^

^1^Aier School of Ophthalmology, Central South University, Changsha, China.

^2^ Changsha Aier Eye Hospital, Changsha, Hunan, China.

^3^ Aier Institute of Optometry and Vision Science, Aier Eye Hospital Group, Changsha, China.

^4^ Aier School of Optometry and Vision Science, Hubei University of Science and Technology, Xianning, Hubei, China.

^5^ Shanghai Aier Eye Hospital, Shanghai, China.

^6^ The Wellcome-Wolfson Institute for Experimental Medicine, School of Medicine, Dentistry and Biomedical Sciences, Queen’s University Belfast, 97 Lisburn Road, Belfast, BT9 7BL, UK.

*** Correspondence:**

Corresponding to Professor Heping Xu, Tel: +44(0)28909 76463, Email: [heping.xu@qub.ac.uk](mailto:heping.xu@qub.ac.uk) or Professor Zhikuan Yang, +86(0)13380071988, Email: [yangzhikuan@aierchina.com](mailto:yangzhikuan@aierchina.com)

1. **Supplementary Tables**

**Supplementary table 1**. Clinical characteristics of patients with high myopia and low myopia.

|  | Low myopia | High myopia | *P* values |
| --- | --- | --- | --- |
|  | (n=60) | (n=87) |  |
| Gender, n (%) |  |  | 0.12§ |
| Females | 50 (83.3%) | 62 (71.3%) |  |
| Males | 10 (16.7%) | 25 (28.7%) |  |
| Age (years) | 26.37 ± 5.08 | 25.78 ± 6.37 | 0.55* |
| Family history of myopia, n (%) |  |  | 1‡ |
| Y | 2 (3.3%) | 3 (3.4%) |  |
| N | 58 (96.7%) | 84 (96.6%) |  |
| History of photocoagulation |  |  | 0.27‡ |
| Y | 0 (0.0%) | 3 (3.4%) |  |
| N | 60 (100.0%) | 84 (96.6%) |  |
| BCVA | 1.00 ± 0.00 | 0.99 ± 0.06 | 0.32* |
| Spherical equivalent (D) | -6.11 ± 1.22 | -10.65 ± 3.42 | **<0.001*** |
| Axial length (mm) | 25.53 ± 0.60 | 27.70 ± 1.53 | **<0.001*** |

§ Chi-square test; ‡ Fisher’s test; * Student’s t-test.

**Supplementary table 2**. Clinical characteristics of patients with different degrees of pathological myopia.

|  | SM (n=78) | PS  (n=39) | PS + CA  (n=30) | *P* values | *P* values  SM vs PS | *P values*  *SM vs* PS + CA | *P* values  PS vs PS + CA |
| --- | --- | --- | --- | --- | --- | --- | --- |
| Gender, n(%) |  |  |  | 0.97§ | 0.82§ | 1§ | 1§ |
| Females | 60 | 29 | 23 |  |  |  |  |
| Males | 18 | 10 | 7 |  |  |  |  |
| Age (years) | 25.88±5.63 | 25.44±5.09 | 27.13±7.29 | 0.47^a^ | 0.68^b^ | 0.35^b^ | 0.28^b^ |
| Family history of myopia, n (%) |  |  |  | 0.71‡ | 1‡ | 0.56‡ | 0.5‡ |
| Y | 3(3.8%) | 2(5.1%) | 0(0.0%) |  |  |  |  |
| N | 75(96.2%) | 37(94.9%) | 37(94.9%) |  |  |  |  |
| History of photocoagulation |  |  |  | 1‡ | 1‡ | 1‡ | 1‡ |
| Y | 2 (2.6%) | 1 (2.6%) | 0 (0.0%) |  |  |  |  |
| N | 76 (97.4%) | 38 (97.4%) | 30 (100.0%) |  |  |  |  |
| BCVA | 1.00±0.00 | 1.00±0.00 | 0.98±0.11 | 0.14^a^ | 1^b^ | 0.33^b^ | 0.33^b^ |
| Spherical equivalent (D) | -7.39±2.10 | -8.86±2.64 | -12.37±4.85 | **<0.001^a^** | **0.001^b^** | **<0.001^b^** | **<0.01^b^** |
| Axial length (mm) | 26.08±0.95 | 27.12±1.59 | 28.33±1.95 | **<0.001^a^** | **<0.001^b^** | **<0.001^b^** | **0.006^b^** |

§Chi-square analysis; ‡ Fisher’s test; ^a^ ANOVA; ^b^ Bonferroni’post hoc analysis; SM: simple myopia; PS: posterior staphyloma; CA: myopic chorioretinal atrophy; PS: posterior staphyloma; PS+CA: posterior staphyloma with myopic chorioretinal atrophy.

**Supplementary table 3.** Descriptive statistics for selected features of the macular zone and optic nerve head in patients with different degrees of pathological myopia.

|  | SM (n=78) | PS  (n=39) | PS + CA  (n=30) | *P* values^a^ | *P* values  SM vs PS^b^ | *P* values  SM vs PS + CA^b^ | *P* values  PS  vs PS + CA^b^ |
| --- | --- | --- | --- | --- | --- | --- | --- |
| **Macular zone** | | | | | | | |
| SRVD (%) | 50.56±2.58 | 50.03±3.29 | 48.15±3.99 | **0.0499** | 0.51 | **0.04** | 0.11 |
| SRT (μm) | 280.00±12.07 | 278.13±18.66 | 266.38±11.25 | **0.009** | 0.67 | **<0.001** | **<0.001** |
| DRVD (%) | 48.88± 4.96 | 46.93± 5.44 | 42.43± 7.12 | **<0.001** | 0.09 | **<0.001** | **0.01** |
| DRT (μm) | 282.54±12.33 | 280.19±17.11 | 264.83±11.37 | **<0.001** | 0.49 | **<0.001** | **<0.001** |
| FAZ (mm^2^) | 0.29±0.09 | 0.25±0.09 | 0.42±0.80 | 0.25 | 0.06 | 0.47 | 0.35 |
| PERIM (mm) | 2.06±0.33 | 1.91±0.35 | 2.32±1.84 | 0.26 | **0.047** | 0.53 | 0.32 |
| FD (%) | 55.70±4.66 | 55.32±4.25 | 47.84±7.18 | **<0.001** | 0.72 | **<0.001** | **0.001** |
| Average GCC (μm) | 96.20±7.10 | 94.16±8.13 | 92.00± 5.39 | 0.15 | 0.29 | 0.049 | 0.38 |
| **Optic nerve head** | | | | | | | |
| Average RNFL (μm) | 97.73±10.18 | 96.96±9.93 | 90.93±11.05 | 0.1 | 0.77 | **0.04** | 0.09 |
| ONHVD-all vessels (%) | 56.03±2.49 | 53.09±4.89 | 52.70±3.83 | **<0.001** | **0.006** | **0.001** | 0.76 |
| ONHVD-small vessels (%) | 49.83±2.52 | 47.23±4.64 | 46.81±3.89 | **0.001** | **0.01** | **0.003** | 0.74 |
| IDVD-all vessels (%) | 61.89±4.02 | 63.27±4.46 | 62.93±3.63 | 0.32 | 0.17 | 0.31 | 0.78 |
| IDVD-small vessels (%) | 54.09±5.10 | 57.55±5.53 | 55.69±4.91 | **0.02** | **0.008** | 0.23 | 0.23 |
| Peripapillary VD-all vessels (%) | 58.02±3.10 | 54.20±6.01 | 53.16±4.96 | **<0.001** | **0.005** | **<0.001** | 0.53 |
| Peripapillary VD-small vessels (%) | 51.85±3.23 | 48.45±5.61 | 47.52±4.88 | **<0.001** | **0.008** | **<0.001** | 0.55 |

^a^ ANOVA; ^b^ Bonferroni’post hoc analysis; *P* value<0.05 was considered statically significant; SM: simple myopia; PS: posterior staphyloma; CA: myopic chorioretinal atrophy; PS: posterior staphyloma; PS+CA: posterior staphyloma with myopic chorioretinal atrophy; SRVD: superficial retinal vessels density; SRT: superficial retinal thickness; DRVD: deep layer retinal vessels density; DRT: deep layer retinal thickness; FAZ: Foveal avascular zone; PERIM: FAZ perimeter; FD: Foveal density; GCC: ganglion cell complex; Average RNFL: average retinal nerve fiber layers; ONHVD-all vessels: optic nerve head all vessel density; ONHVD-small vessels: optic nerve head small vessel density; IDVD-all vessels: inside disc all vessel density; IDVD- small vessels: inside disc small vessel density; Peripapillary VD-all vessels: Peripapillary all vessel density; Peripapillary VD-small vessels: Peripapillary small vessel density.

**Supplementary table 4**. The correlations between intraocular complement levels and SRT in the macular zone

|  | Group(n=147) | | SM(n=78) | | PS(n=39) | | PS+CA(n=30) | |
| --- | --- | --- | --- | --- | --- | --- | --- | --- |
|  | β(SE) | *P* values† | β(SE) | *P* values† | β(SE) | *P* values† | β(SE) | *P* values† |
| **Complement components/fragments of the CP** | | | | | | | | |
| C1q | -0.14 (0.04) | **<0.001** | 0.02 (0.05) | 0.74 | -0.09 (0.04) | **0.048** | -0.57 (0.16) | **0.003** |
| C2 | -0.03 (0.01) | **0.02** | -0.00 (0.01) | 0.68 | -0.01 (0.01) | 0.44 | -0.07 (0.06) | 0.27 |
| C4 | -1.47 (0.59) | **0.01** | 0.45 (0.91) | 0.62 | -1.20 (0.88) | 0.19 | -3.87 (1.89) | 0.06 |
| C4b | -0.52 (0.20) | **0.01** | -0.09 (0.29) | 0.76 | -0.36 (0.27) | 0.21 | -0.99 (0.85) | 0.26 |
| **Complement components/fragments of the AP** | | | | | | | | |
| CFB | -0.49 (0.28) | 0.09 | 0.90 (0.45) | 0.054 | -0.24 (0.38) | 0.54 | -3.03 (0.63) | **<0.001** |
| CFH | -0.30 (0.12) | **0.01** | 0.20 (0.18) | 0.28 | -0.33 (0.21) | 0.13 | -1.14 (0.27) | **<0.001** |
| CFI | -0.54 (0.25) | **0.03** | 0.76 (0.38) | 0.055 | -0.21 (0.31) | 0.51 | -3.10 (0.53) | **<0.001** |
| CFD | -0.10 (0.07) | 0.19 | 0.07 (0.12) | 0.56 | -0.04 (0.13) | 0.76 | -0.63 (0.14) | **<0.001** |
| **Lectin Pathway** | | | | | | | | |
| MBL | 0.00 (0.00) | 0.42 | -0.00 (0.00) | 0.83 | 0.00 (0.00) | 0.39 | -0.00 (0.00) | 0.43 |
| **Complement components/fragments of the shared pathway** | | | | | | | | |
| C3 | -0.84 (0.34) | **0.02** | 0.25 (0.61) | 0.68 | -1.02 (0.51) | 0.06 | -2.77 (0.88) | **0.007** |
| C3b/iC3b | -1.65 (0.49) | **0.001** | 0.30 (0.67) | 0.66 | -1.08 (0.65) | 0.11 | -6.08 (1.57) | **0.002** |
| C5 | -0.03 (0.02) | 0.15 | -0.02 (0.03) | 0.41 | 0.01 (0.03) | 0.61 | -0.02 (0.06) | 0.73 |

β: regression coefficient; SE: standard error; † Unadjusted Linear Regression; SM: simple myopia; PS: posterior staphyloma; CA: myopic chorioretinal atrophy; PS: posterior staphyloma; PS+CA: posterior staphyloma with myopic chorioretinal atrophy.

**Supplementary table 5**. The correlations between intraocular complement levels and SRVD in the macular zone

|  | All (n=147) | | SM (n=78) | | PS (n=39) | | PS+CA (n=30) | |  | |
| --- | --- | --- | --- | --- | --- | --- | --- | --- | --- | --- |
|  | β(SE) | *P* values† | β(SE) | *P* values† | β (SE) | *P* values† | β(SE) | *P* values† | |  |
| **Complement components/fragments of the CP** | | | | | | |  | |  |  |
| C1q | -0.64 (0.19) | **0.001** | 0.09 (0.23) | 0.68 | -0.63 (0.24) | **0.01** | -1.05 (0.55) | | 0.08 | |
| C2 | -0.09 (0.06) | 0.09 | -0.01 (0.05) | 0.85 | -0.11 (0.07) | 0.10 | 0.01 (0.17) | | 0.96 | |
| C4 | -4.38 (2.81) | 0.12 | 3.84 (4.20) | 0.37 | -8.83 (4.82) | 0.08 | -0.92 (6.07) | | 0.88 | |
| C4b | -0.76 (0.99) | 0.44 | 1.00 (1.34) | 0.46 | -1.91 (1.56) | 0.23 | 1.25 (2.48) | | 0.62 | |
| **Complement components/fragments of the AP** | | | | | | |  | |  |  |
| CFB | -2.97 (1.31) | **0.03** | 1.30 (2.23) | 0.56 | -3.80 (2.03) | 0.07 | -3.15 (2.77) | | 0.27 | |
| CFH | -1.03 (0.57) | 0.08 | 0.67 (0.85) | 0.43 | -1.78 (1.18 | 0.14 | -1.14 (1.11) | | 0.32 | |
| CFI | -1.69 (1.19) | 0.16 | 1.64 (1.87) | 0.39 | -0.35 (1.77) | 0.85 | -2.99 (2.66) | | 0.28 | |
| CFD | -0.29 (0.35) | 0.40 | 0.74 (0.56) | 0.19 | -0.60 (0.71) | 0.41 | -0.61 (0.60) | | 0.33 | |
| **Lectin Pathway** | | | | | | |  | |  |  |
| MBL | 0.00 (0.00) | 0.17 | 0.00 (0.01) | 0.95 | 0.01 (0.01) | 0.22 | 0.00 (0.00) | | 0.63 | |
| **Complement components/fragments of the shared pathway** | | | | | | |  | |  |  |
| C3 | -1.80 (1.64) | 0.28 | 4.18 (2.76) | 0.14 | -4.70 (3.00) | 0.13 | -3.14 (3.13) | | 0.33 | |
| C3b/iC3b | -7.60 (2.27) | **0.001** | 1.82 (3.15) | 0.57 | -8.90 (3.40) | **0.02** | -10.14 (5.77) | | 0.10 | |
| C5 | -0.15 (0.09) | 0.11 | 0.06 (0.13) | 0.62 | -0.15 (0.15) | 0.35 | -0.04 (0.18) | | 0.84 | |

β: regression coefficient; SE: standard error; † Unadjusted Linear Regression; SM: simple myopia; PS: posterior staphyloma; CA: myopic chorioretinal atrophy; PS: posterior staphyloma; PS+CA: posterior staphyloma with myopic chorioretinal atrophy; SRVD: superficial retinal vessel density.

**Supplementary table 6**. The correlation between intraocular complement levels and average RNFL in the optic nerve head.

|  | All (n=147) | | SM (n=78) | | PS (n=39) | | PS+CA (n=30) | |
| --- | --- | --- | --- | --- | --- | --- | --- | --- |
|  | β(SE) | *P* values† | β(SE) | *P* values† | β(SE) | *P* values† | β(SE) | *P* values† |
| **Complement components/fragments of the CP** | | | | | | | | |
| C1q | -0.14 (0.06) | **0.02** | 0.03 (0.04) | 0.44 | -0.14 (0.09) | 0.13 | -0.42 (0.23) | 0.09 |
| C2 | -0.01 (0.01) | 0.28 | 0.01 (0.01) | 0.55 | -0.03 (0.02) | 0.27 | 0.00 (0.04) | 0.91 |
| C4 | -0.75 (0.86) | 0.38 | 1.49 (0.95) | 0.13 | -2.67 (1.72) | 0.14 | -1.42 (2.37) | 0.56 |
| C4b | -0.12 (0.29) | 0.69 | 0.38 (0.33) | 0.26 | -0.55 (0.57) | 0.34 | -0.02 (0.92) | 0.99 |
| **Complement components/fragments of the AP** | | | | | | | | |
| CFB | -0.13 (0.41) | 0.76 | 1.26 (0.47) | **0.01** | -0.77 (0.64) | 0.24 | -1.28 (1.16) | 0.29 |
| CFH | -0.04 (0.18) | 0.82 | 0.49 (0.19) | **0.01** | -0.30 (0.41) | 0.47 | -0.56 (0.45) | 0.24 |
| CFI | -0.31 (0.35) | 0.38 | 0.50 (0.38) | 0.2 | -0.10 (0.57) | 0.86 | -1.39 (1.15) | 0.25 |
| CFD | 0.07 (0.12) | 0.53 | 0.28 (0.16) | 0.08 | 0.01 (0.24) | 0.98 | -0.14 (0.23) | 0.56 |
| **Lectin Pathway** | | | | | | | | |
| MBL | 0.00 (0.00) | **0.003** | 0.01 (0.00) | **0.004** | 0.00 (0.00) | 0.38 | 0.00 (0.00) | 0.82 |
| **Complement components/fragments of the shared pathway** | | | | | | | | |
| C3 | -0.25 (0.50) | 0.62 | 1.23 (0.68) | 0.08 | -2.61 (0.66) | **<0.001** | -0.13 (1.40) | 0.93 |
| C3b/iC3b | -1.17 (0.68) | 0.09 | 1.52 (0.54) | **0.008** | -1.39 (1.18) | 0.25 | -5.09 (2.20) | **0.04** |
| C5 | -0.02 (0.03) | 0.37 | -0.02 (0.03) | 0.5 | 0.00 (0.05) | 0.96 | 0.03 (0.07) | 0.65 |

β: regression coefficient; SE: standard error; † Unadjusted Linear Regression; SM: simple myopia; PS: posterior staphyloma; CA: myopic chorioretinal atrophy; PS: posterior staphyloma; PS+CA: posterior staphyloma with myopic chorioretinal atrophy; RNFL: retinal nerve fiber layer.

**Supplementary table 7**. The correlation between intraocular complement levels and peripapillary VD-all vessels in the optic nerve head.

|  | All (n=147) | | SM (n=78) | | PS (n=39) | | PS+CA (n=30) | |
| --- | --- | --- | --- | --- | --- | --- | --- | --- |
|  | β(SE) | *P* values† | β(SE) | *P* values† | β(SE) | *P* values† | β(SE) | *P* values† |
| **Complement components/fragments of the CP** | | | | | | | | |
| C1q | **-0.40 (0.12)** | **0.002** | **0.30 (0.16)** | **0.06** | **-0.08 (0.14)** | **0.55** | **-0.98 (0.40)** | **0.02** |
| C2 | **-0.10 (0.03)** | **0.003** | **0.06 (0.03)** | **0.051** | **-0.08 (0.04)** | **0.04** | **-0.09 (0.12)** | **0.45** |
| C4 | **-3.37 (1.65)** | **0.04** | **5.40 (2.97)** | **0.08** | **-3.02 (2.68)** | **0.27** | **-0.62 (3.70)** | **0.87** |
| C4b | -0.68 (0.55) | 0.22 | 1.25 (0.91) | 0.18 | -0.60 (0.85) | 0.48 | 0.60 (1.52) | 0.70 |
| **Complement components/fragments of the AP** | | | | | | | | |
| CFB | -2.90 (0.77) | **<0.001** | 3.28 (1.51) | **0.04** | -2.36 (1.07) | **0.04** | -4.45 (1.52) | **0.009** |
| CFH | -0.75 (0.39) | 0.055 | 2.06 (0.62) | **0.002** | -0.28 (0.67) | 0.68 | -2.37 (0.86) | **0.01** |
| CFI | -1.39 (0.68) | **0.04** | 2.07 (1.13) | 0.08 | -0.93 (0.86) | 0.29 | -3.55 (1.94) | 0.08 |
| CFD | -0.25 (0.21) | 0.24 | 1.01 (0.40) | **0.01** | -0.29 (0.34) | 0.42 | -0.85 (0.47) | 0.09 |
| **Lectin Pathway** | | | | | | | | |
| MBL | -0.00 (0.00) | **0.04** | 0.00 (0.00) | 0.28 | -0.00 (0.00) | 0.33 | -0.01 (0.01) | 0.11 |
| **Complement components/fragments of the shared pathway** | | | | | | | | |
| C3 | -1.54 (0.96) | 0.11 | 6.08 (1.82) | **0.002** | -2.70 (1.69) | 0.12 | -3.27 (1.87) | 0.10 |
| C3b/iC3b | -5.72 (1.52) | **<0.001** | 4.46 (1.61) | **0.008** | -1.79 (1.93) | 0.36 | -13.44 (4.49) | **0.007** |
| C5 | -0.18 (0.05) | **<0.001** | -0.06 (0.07) | 0.42 | -0.14 (0.07) | 0.06 | -0.03 (0.13) | 0.79 |

β: regression coefficient; SE: standard error; † Unadjusted Linear Regression; SM: simple myopia; PS: posterior staphyloma; CA: myopic chorioretinal atrophy; PS: posterior staphyloma; PS+CA: posterior staphyloma with myopic chorioretinal atrophy; VD: vessel density.

.

**Supplementary table 8**. **Primer sequence used in the study.**

| **Target genes** | **Forward** | **Reverse** |
| --- | --- | --- |
|  |  |  |
| *C1q* | CAAGGTGGTGGTCTTCGACA | CTATCCAGACCTTGTCGCCC |
| *C2* | TGTCAGCCAATGCCTCTGAC | CAGGATCCCCCACGATGAC |
| *C3* | GTCACCTCCCCCTACCAGAT | GTGAGAGACTGCACGTTGGA |
| *C4(C4a)* | TGCGGTGTCCAAGGTTCTAC | ACAAAGCTGCTCGACTCTCC |
| *CFD* | CACCATCGATCACGACCTCC | CCACATCGCAGAGAGTCCC |
| *CFI* | GGGCTCTTAGAAACAGCAGC | CTCAGGTCTTCTGACGCCAC |
| *Actb* | CGAGTACATCCCCTCGCTTC | TTTTGCTCTGGGCTTCGTCT |

1. [↑](#footnote-ref-1)
